# Incidental low grade mucinous neoplasm of appendix in pregnancy: A case report & literature review

**DOI:** 10.1016/j.amsu.2020.10.004

**Published:** 2020-10-07

**Authors:** Nitasha Saleem, Fakhar Shahid, Syed Mohammed Ali, Sameera Rashid, Mohannad Al-Tarakji, Mohammad Sameer

**Affiliations:** a Department of General Surgery, Hamad Medical Corporation, Doha, Qatar; b Department of Acute Care Surgery, Hamad Medical Corporation, Doha, Qatar; c Department of Pathology, Hamad Medical Corporation, Doha, Qatar

**Keywords:** Acute abdomen, Appendicitis, Pregnant, Mucinous cancer, Case report

## Abstract

**Introduction:**

Mucinous neoplasms of appendix account for 0.2–0.4% of all the appendix specimens. The occurrence of this neoplasm in pregnancy is extremely rare. We describe a case of a pregnant lady who was diagnosed as acute appendicitis and found to have Low-Grade Mucinous neoplasm on histopathology. In the existent literature, there are only a few such cases reported and none from our Middle East region.

**Case presentation:**

42-year-old pregnant lady at 24 weeks of gestation presented with classical symptoms of acute appendicitis. She had leukocytosis but the Ultrasound was equivocal. She underwent laparoscopic appendectomy and found to have an inflamed appendix. Postoperative recovery was satisfactory and was discharged home. The histopathology report showed low-grade mucinous neoplasm of the appendix and she was detailed about it on follow up.

**Discussion:**

The incidence of appendiceal neoplasm is rare in routine appendectomy and carcinoid is the most common tumor of the appendix. Low-Grade mucinous neoplasm is a rare entity and its presence in pregnancy is further rarer.

**Conclusion:**

Since this neoplasm does not manifest with a characteristic clinical profile it is difficult to diagnose, even with extensive preoperative evaluation. Although surgical treatment is straight forward, the management of the appendiceal neoplasm during pregnancy necessitates full knowledge of the natural history of the disease to attain equilibrium of concern for maternal survival and fetal health.

## Highlights

• Mucinous neoplasms of appendix are known to be rarely found in all the appendix specimens, but the occurrence of this neoplasm in pregnancy is extremely rare.
• This neoplasm does not manifest with distinctive clinical profile and it is challenging to diagnose it, even with extensive preoperative evaluation.
• It is questionable to decide whether to allow pregnancy or terminate unless proper work up is not done to decide the nature of this neoplasm.
• Further studies dedicated to the understanding of the mucinous neoplasms can help us with designing a more targeted therapeutic plan for such patients.

## 1 Introduction

Appendiceal mucinous neoplasms (AMNs) are an uncommon group of tumors. Incidence of 0.2%–0.4% is reported among all patients who have undergone an appendectomy, and usually, it occurs in patients aged 50–60 years [1,2] LAMNs have diverse histology and can be classified as colonic-type, mucinous adenocarcinoma, goblet cell adenocarcinoma, or neuroendocrine carcinoma [3].

Since mucinous neoplasms are a rare cause of acute abdomen, they are often diagnosed as acute appendicitis initially. Especially, in younger patients, due to a low threshold for the diagnosis of acute appendicitis rarely a preoperative diagnosis is made [4]. Computed Tomography (CT) scan is the diagnostic modality that aids in preoperative diagnosis as it can detect appendiceal mucocele or even pseudomyxoma peritonei [4]. A preoperative diagnosis sets a precautious approach for the surgeon to avoid accidental iatrogenic perforation of the appendix during surgery to avoid the development of pseudomyxoma peritonei, which is the most feared complication characterized by peritoneal dissemination with high morbidity and mortality rate [4]. This work has been reported in line with the SCARE 2018 criteria [5].

## 2 Case Presentation

A 42-year-old female (gravida 4, para 2, abortion 1) at 24 weeks of gestation, presented to the Emergency Department with the complaint of right iliac fossa pain of 8 h duration. It was gradual in onset, progressive, severe in intensity, and non-radiating without nausea, vomiting, anorexia, or fever. She had no past medical history and she has never had any surgical procedure in the past. She did not take any drugs or medications. She did not smoke but takes alcohol occasionally. Her family history is negative for any malignancy. On examination she was tender on palpation in right iliac fossa with no rigidity or guarding but with rebound tenderness. Her WBC count was high (13.8 × 10^3^/μl) with increased neutrophil count and high lactic acid of 3.3mmol/L. US abdomen did not show the appendix per se due to gassy abdomen, but minimal fluid was seen in right iliac fossa with viable intrauterine fetus.

**Fig. 1:**
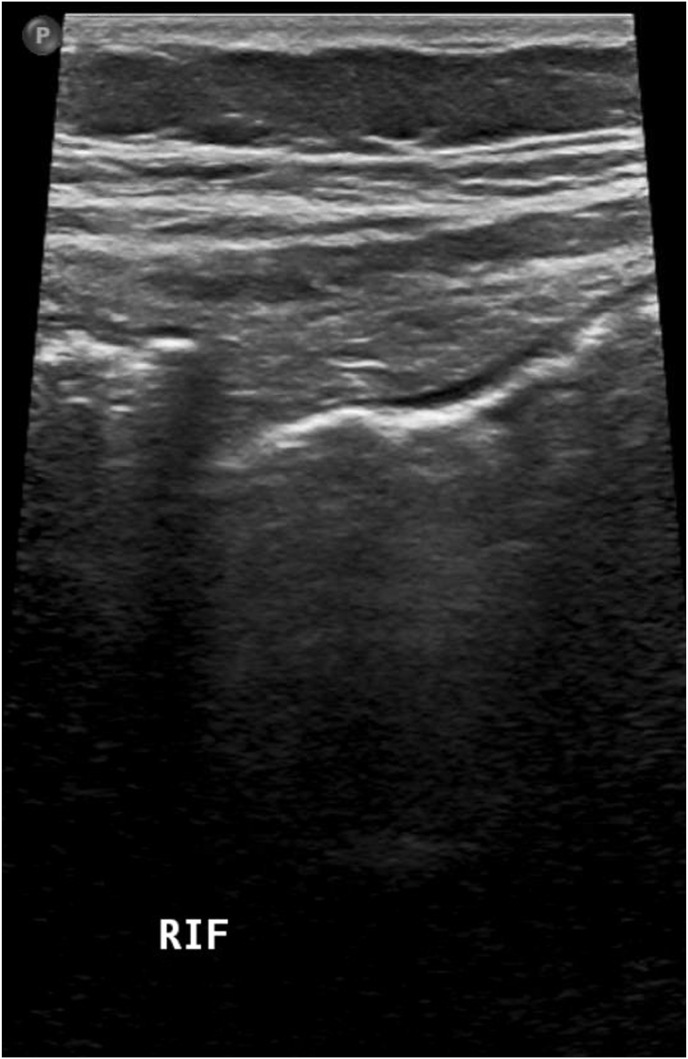
US image of Right Iliac fossa with equivocal findings due to gassy abdomen.

**Fig. 2:**
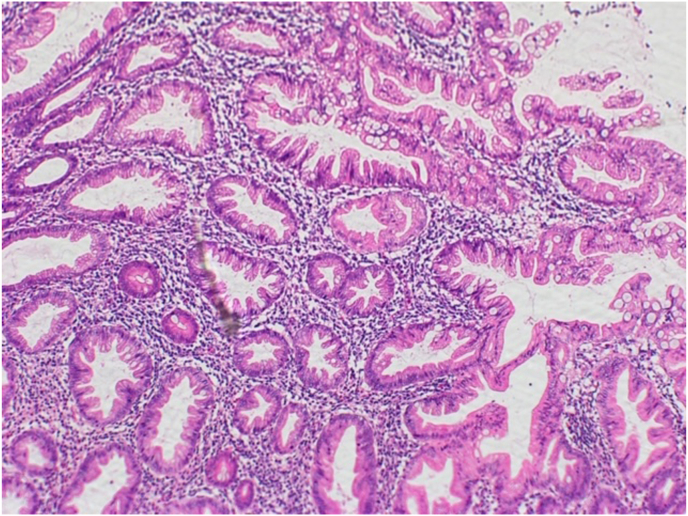
Hemotoxylin and Eosin stained section showing Epithelial dysplasia with mucinous change.

**Fig. 3:**
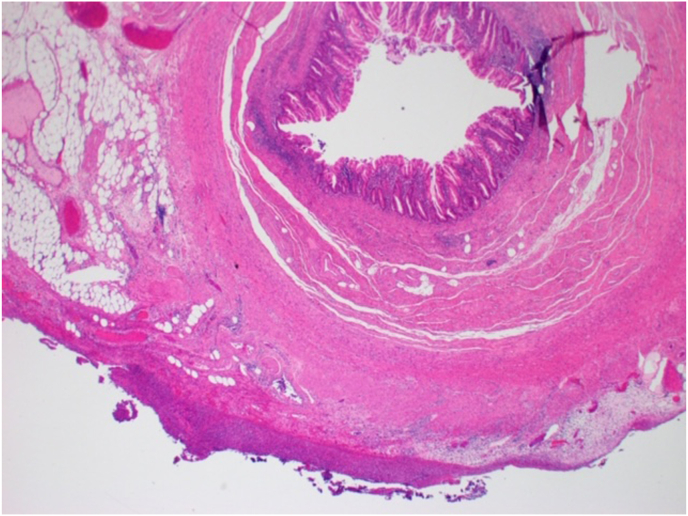
Low power view showing transmural inflammation.

**Fig. 4:**
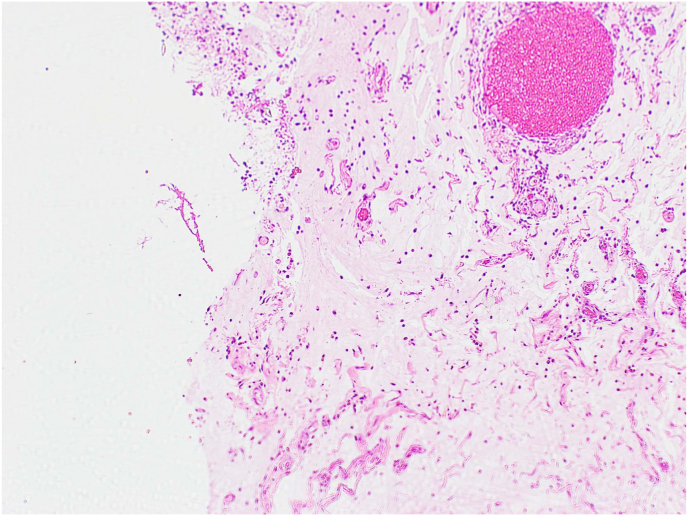
High power view of the appendix surface shows acellular mucin].

She was observed for 24 hours but her pain and tenderness increased significantly, and she underwent diagnostic laparoscopy. The Surgery was performed by a middle grade Surgeon specialist under the supervision of the Attending. Intraoperatively, the appendix was found to be thickened and almost 1 cm in diameter with severe inflammation. The appendix was ligated at the base using endoloop and cut near the base. After that the appendix was safely placed in the endobag and removed from the body. She had an uneventful recovery post-surgery. She was discharged home after 2 days without any concerns. Histopathology reported acute appendicitis and low grade mucinous appendiceal neoplasm of about 1.4 cm near the tip of appendix. There were no lymph nodes present. Dysplastic changes were confined to the mucosa whereas resection margin was free (See, Fig. 1, Fig. 2, Fig. 3, Fig. 4).

On her follow up visit after 2 weeks from surgery she was seen in clinic and found healthy with no adverse effects from surgery. She was informed about her pathology results. She received the news with a shock, but she was reassured. The Surgery team never suspected this outcome. She was suggested to follow up for further investigations and treatment. Her case was discussed in oncology multidisciplinary meeting. It was concluded that she should be further investigated with MRI pelvis & colonoscopy and be offered HIPEC. She underwent an uneventful normal vaginal delivery at 40 weeks gestational age. Post-delivery, she resumed follow-up. She underwent further imaging where her colonoscopy result was normal and MRI pelvis showed no intraperitoneal metastasis. Patient was offered treatment option of HIPEC which she refused. She came for follow up in the clinic for 10 months but after that she stopped coming to the appointments and lost follow up.

## 3 Discussion

LAMNs are a rare pathology of the appendix [6]. To our knowledge, there are only a few previous reports of a LAMN occurring during pregnancy [7]. LAMNs are tumors localized in the appendix or the surrounding appendiceal mucosa wall [6]. These tumors characteristically cause cystic dilatation of the appendix owing to the accumulation of copious gelatinous material in the lumen. They may disseminate throughout the peritoneal cavity in the form of gelatinous deposits, termed pseudomyxoma peritonei (PMP) [8].

The first classification of mucinous neoplasm was made by Misdraji et al. into low-grade appendiceal mucinous neoplasms (LAMNs) and mucinous adenocarcinoma based on the complexity of architecture and degree of cytological atypia in 2003 [9], which was adopted later by the World Health Organization classification in 2010 (See, Table 1).

**Table 1:** Classification of Mucinous neoplasms.

Pathological features	Misdraji et al.	World Health Organization
Tumors without invasion (intact muscularis mucosae)	Low-grade appendiceal mucinous neoplasm	Adenoma
Tumors with pushing invasion Tumor confined to the appendix	Low-grade appendiceal mucinous neoplasm	Low-grade appendiceal mucinous neoplasm
Tumor with acellular mucin outside the appendix	Low-grade appendiceal mucinous neoplasm	Low-grade appendiceal mucinous neoplasm
Tumor with extra-appendiceal tumor	Low-grade appendiceal mucinous neoplasm	Low-grade appendiceal mucinous neoplasm
Tumors with infiltrative invasion Infiltrative type invasion of the wall, with or without desmoplasia, regardless of stage	Mucinous Adenocarcinoma	Mucinous Adenocarcinoma

As is the incidence with most neoplasms of the appendix and our case, the most common presentation is with abdominal pain similar to that of acute appendicitis. This may be from distention of the appendix causing pain or from a superinfection [10]. Carr et al. reported that 32% of patients with appendiceal neoplasms received a preoperative diagnosis of acute appendicitis, while 23% were incidentally diagnosed [11]. These neoplasms are more commonly diagnosed in men, particularly in the sixth decade [7]. Diagnostic imaging modalities that are frequently used are Ultrasound and abdominal CT. Ultrasound can show a mass with small echo spots and/or a concentric echo layer (known as the “onion skin”), both of which have been suggested as ANM-specific changes [12]. According to one of the other studies, the diagnosis for AMN has a sensitivity of 83% and a specificity of 92% when the diameter of the ultrasound-visualized appendix exceeds 15 mm [2]. However, the most useful imaging method is abdominal CT [13], as it can evaluate the relationship between the formation of peripheral organs, which may make diagnosis easier. When CT findings are cystic structures closely related to the cecum with round or long tubular shape, thin cyst wall, and smooth outline, the possibility of appendiceal mucinous adenocarcinoma should be considered; irregular cyst wall and soft tissue thickening are also suggestive features of malignancy. The finding of appendix cavity diameter exceeding 13 mm prompts high suspicion of AMN [14]. In few instances, a colonoscopy might be used as an imaging modality and an important indication found during colonoscopy is the so-called “volcano sign” - a visible raised zone in the cecum, with an appendicular orifice located in its center [11].

Our case is unique because pre- and intra-operative diagnosis was acute appendicitis as the age was 42; female, no feature of malignancy on presentation, and since neoplastic disease concomitant with pregnancy is fortunately rare. But histopathology confirmed it to be low-grade mucinous neoplasm of the appendix. Therefore, histopathological examination of all appendectomy specimens is mandatory to rule out malignant pathology.

The treatment with the most curative potential is surgery. Our patient underwent a laparoscopic procedure that allows a better evaluation of the abdominal cavity and rapid patient recovery. Unfortunately, the laparoscopic approach carries the increased risk of rupture of the mucocele and provocation of a PMP [4]. Based on our case and the previous case reports, it appears reasonable to carry out a diagnostic surgical evaluation of a mucinous appendiceal tumor during pregnancy, ideally in the second or third trimester [15]. For patients with LAMN, prophylactic CRS + HIPEC treatment can achieve the greatest survival benefit, but there is an unknown risk of overtreatment [16]. Keeping this evidence in view we offered our patient HIPEC, which she refused. We also know that the treatment of LAMN is under debate, but many authors are using the HIPEC in their management. Due to the young age of our patient, pathology findings, and in the light of the published literature our MDT decided the above-mentioned management for this patient.

Further studies dedicated to the understanding of the mucinous neoplasms can help us with designing a more targeted therapeutic plan for such patients. For pregnant patients with the slow or moderate advance of the disease, the pregnancy (or pregnancy wish) should be allowed to proceed to vaginal delivery. In patients with rapid progression, termination of the pregnancy, and definitive treatment may be necessary to protect the mother [15].

## 4 Conclusion

Low-Grade Mucinous neoplasm of the appendix is a rare neoplasm. The occurrence of this neoplasm in pregnancy is extremely erratic. It is very challenging to diagnose it preoperatively as it does not present in a typical manner, even with extensive preoperative evaluation. Further studies are needed to understand the management of such incidental lesions and the impending complications of a devastating peritoneal disease. Particularly, during pregnancy full knowledge of the natural history of this disease is required to attain equilibrium of concern for maternal survival and fetal health.

### 4.1 Informed consent

Written informed consent was obtained from the patient for publication of this case report and accompanying images. A copy of the written consent is available for review by the Editor-in-Chief of this journal on request.

## Funding

No Funding was applied for this article.

## Ethical committee approval

The Study was approved by the Medical Research Committee of Hamad Medical Corporation for publication and the reference number is MRC-04-20-665.

## Provenance and peer review

Not commissioned, externally peer reviewed.

## Author contribution

Nitasha Saleem: Data collection, , Manuscript Writing and Review.

Fakhar Shahid: Study concept, Data interpretation and literature review.

Syed Muhammed Ali: Data interpretation and manuscript writing.

Sameera Rashid: Histopathology slides and review.

Mohannad Al-Tarakji: Concept design and literature review.

Mohammad Sameer: Manuscript editing and collection of images.

## Declaration of competing interest

Authors have no Conflict of interest to be declared.

## Abbreviations

LAMN: low-grade mucinous neoplasm
MDT: Multidisciplinary team
HIPEC: hyper-thermic intraperitoneal chemotherapy

## References

[1] Zagrodnik D.F., Rose D.M. (2003). Mucinous cystadenoma of the appendix: diagnosis, surgical management, and follow-up. Curr. Surg..

[2] Karakaya K., Barut F., Emre A.U., Ucan H.B., Cakmak G.K., Irkorucu O., Tascilar O., Ustundag Y., Comert M. (2008). Appendiceal mucocele: case reports and review of current literature. World J. Gastroenterol..

[3] Kelly K.J. (2015). Management of appendix cancer. Clin. Colon Rectal Surg..

[4] Bennett G.L., Tanpitukpongse T.P., Macari M., Cho K.C., Babb J.S. (2009). CT diagnosis of mucocele of the appendix in patients with acute appendicitis. Am. J. Roentgenol..

[5] Agha Riaz A., Borrelli Mimi R., Farwana Reem, Koshy Kiron, Alexander J., Fowler, Dennis P. (2018). Orgill, for the SCARE group. The SCARE 2018 statement: updating consensus surgical CAse REport (SCARE) guidelines. Int. J. Surg..

[6] Ramaswamy V. (2016). Pathology of mucinous appendiceal tumors and pseudomyxoma peritonei. Indian J Surg Oncol.

[7] Inubashiri E., Watanabe Y., Akutagawa N., Kuroki K., Sugawara M., Deguchi K., Maeda N., Hata F. (2020). An incidental finding of low‐grade appendiceal mucinous neoplasm during cesarean section: a case report. JGH Open.

[8] Panarelli N.C., Yantiss R.K. (2011). Mucinous neoplasms of the appendix and peritoneum. Arch. Pathol. Lab Med..

[9] Misdraji J., Yantiss R.K., Graeme- Cook F.M. (2003). Appendiceal mucinous neoplasms: a clinicopathologic analysis of 107 cases. Am. J. Surg. Pathol..

[10] Bradley R.F., Stewart J.H., Russell G.B. (2006). Pseudomyxoma peritonei of appendiceal origin: a clinicopathologic analysis of 101 patients uniformly treated at a single institution, with literature review. Am. J. Surg. Pathol..

[11] Carr N.J., McCarthy W.F., Sobin L.H. (1995). Epithelial noncarcinoid tumors and tumor-like lesions of the appendix. A clinicopathologic study of 184 patients with a multivariate analysis of prognostic factors. Cancer.

[12] Garg P.K., Prasad D., Aggarwal S., Mohanty D., Jain B.K. (2011). Acute intestinal obstruction: an unusual complication of mucocele of appendix. Eur. Rev. Med. Pharmacol. Sci..

[13] Rymer B., Forsythe R.O., Husada G. (2015). Mucocoele and mucinous tumours of the appendix: a review of the literature. Int. J. Surg..

[14] Shiihara M., Ohki T., Yamamoto M. (2017). Preoperative diagnosis and surgical approach of appendiceal mucinous cystadenoma: usefulness of volcano sign. Case Rep Gastroenterol.

[15] Haase Erika, Yoo Dal, Sugarbaker Paul (2009). Management of appendiceal pseudomyxoma peritonei diagnosed during pregnancy. World J. Surg. Oncol..

[16] Honoré C., Caruso F., Dartigues P., Benhaim L., Chirica M., Goéré D., Elias D. (2015). Strategies for preventing pseudomyxoma peritonei after resection of a mucinous neoplasm of the appendix. Anticancer Res..

